# Optically Transparent Metamaterial Absorber for Shielding Mechanism Conversion of Satellite Solar Panel

**DOI:** 10.3390/ma19214703

**Published:** 2026-11-05

**Authors:** Shuying Li

**Affiliations:** College of Physics and Electronic Information, Jiangsu Second Normal University, Nanjing 210013, China; lsy527@jssnu.edu.cn

**Keywords:** solar panel, electromagnetic purification, radar stealth, optically transparent metamaterial absorber

## Abstract

**Highlights:**

- Optically transparent metamaterial absorber for satellite solar panel.
- Achieves >90% absorption across 7.63–18.71 GHz (X and Ku bands).
- Converts solar panel shielding from reflection-type to absorption-type.
- Reflection shielding effectiveness drops from >15 dB to <3.01 dB.
- Average visible transmittance reaches 73%.

**Abstract:**

Satellite solar panel inherently produce strong radar echoes and cause secondary electromagnetic pollution due to their highly reflective nature. To solve this, an optically transparent metamaterial absorber (OTMMA) is constructed using the solar panel as the substrate. The proposed multilayer structure consists of a patterned indium tin oxide (ITO) layer, a transparent polymethyl methacrylate (PMMA) spacer, and the solar panel itself. In the 8–18 GHz range, the design achieves over 90% absorptance while maintaining an average visible transmittance of approximately 73%. More importantly, the reflection shielding effectiveness (SE_R_) is reduced from over 15 dB to below 3.01 dB, successfully shifting the shielding mechanism from reflection-dominated to absorption-dominated. Benefiting from this conversion, simulations under normal-incidence and x-polarized conditions demonstrate that the monostatic radar cross section (RCS) of a finite plate is significantly suppressed, achieving a reduction of more than 10 dB across the operating band, with a peak reduction of 20.43 dB at 10.7 GHz. This transforms satellite solar panel from a harmful “electromagnetic pollution source” (strong reflector) into a “functional surface for electromagnetic purification” (optically transparent metamaterial absorber).

## 1. Introduction

With the orderly advancement of space-based reconnaissance radar programs, competition for space dominance has quietly begun, posing serious survivability threats to satellites in orbit [1,2]. Satellite protection has been gradually strengthened in recent years, and the low radar detectability of satellites has become a key technology for improving their survivability [3,4]. Satellite solar panel are important energy-supply components. However, their large physical size and metallized structures readily produce strong radar scattering echoes. As the largest protruding structure, the solar array is a main contributor to the spacecraft radar cross section (RCS) [5]. What makes it worse is that their metal-like characteristics make them reflective electromagnetic shielding materials, causing additional electromagnetic pollution and interfering with onboard electronic systems.

The key to radar stealth is reducing the RCS, primarily through shape stealth and material stealth [6,7]. Traditional absorbing materials generally suffer from narrow bandwidths, poor impedance matching, and optical opacity [8,9,10,11]. More importantly, their design intent is to reduce target echo rather than eliminate the incident waves. Thus, their shielding mechanism remains reflection-dominated, making it difficult to fundamentally solve the secondary pollution problem [12].

Electromagnetic shielding materials are generally classified into reflection-type and absorption-type [13,14]. Reflection-type shields block waves through impedance mismatch, redirecting energy and causing secondary pollution [15]. In contrast, absorption-type shields dissipate incident waves as heat, achieving electromagnetic purification [16]. Recently, researchers have focused on absorption-dominant shields to mitigate secondary pollution [17]. Rong et al. established a theoretical framework for evaluation criteria [18], and Roy et al. emphasized that eco-friendly shields require an SE_R_ well below 3 dB [19]. For millimeter-wave applications, Saeedi et al. highlighted that low-reflectivity absorption-dominant materials are essential [20].

These studies, however, mainly target ground-based applications. Although optically transparent absorbers have been applied to solar arrays [21,22], they mostly focus on bandwidth and transmittance. For instance, Kong et al. [22] attached a transparent absorber to a solar array, but their asymmetric design lacked polarization insensitivity, and their analysis stopped at absorption performance without addressing whether the intrinsic shielding mechanism had changed. Due to their metal-like characteristics, solar panel are typical reflective shields. The criterion distinguishing reflection-type from absorption-type is whether SE_R_ < 3.01 dB [23,24].

Bridging ground-based designs to satellite requirements remains challenging. Most existing shields fail to verify the SE_R_ < 3.01 dB criterion, limiting their ability to truly shift to absorption-dominated shielding [25,26]. Furthermore, achieving simultaneous X- and Ku-band coverage [27,28] while maintaining optical transparency [29,30] is rarely reported. Consequently, a transparent shield that fundamentally converts the solar panel’s reflection mechanism is highly desired.

Metamaterials provide a breakthrough path [31,32]. Since Landy et al. proposed the perfect metamaterial absorber [33], significant progress has been made in understanding their underlying structures and principles [34]. Recent studies have demonstrated multi-band terahertz absorbers using graphene-based resonators [35], while Mo et al. [36] and Wang et al. [37] developed ultra-broadband transparent absorbers utilizing multilayer configurations and laser-etched ITO micro-meshes, respectively. For microwave satellite applications, Zheng et al. designed a transparent absorber covering 6.2–24.6 GHz using multilayer ITO films [38], and Zhao et al. developed an ultra-wideband flexible absorber for satellite stealth [39]. These works achieve impressive bandwidths and transparency through sophisticated structural designs. However, their analyses typically assume ideal metallic grounds and focus primarily on radar stealth or flexibility, leaving the quantitative evaluation of the EMI shielding mechanism largely unexplored.

To fill this gap, this work redefines the solar panel not as a target to be covered, but as a substrate whose shielding mechanism can be fundamentally converted. We construct a realistic multi-layer solar panel model (including Ag grid, GaAs, Ge, and honeycomb core) and rigorously prove its electromagnetic equivalence to an aluminum plate. By integrating a circular OTMMA, we achieve the SE_R_ < 3.01 dB criterion, transforming the panel from a reflection-dominated pollution source into an absorption-dominant purifying surface. The design achieves over 90% absorption in the X- and Ku-bands and maintains a visible transmittance of approximately 73%. Furthermore, simulations performed on a finite 300 × 300 mm^2^ plate under normal-incidence, x-polarized conditions confirm an RCS reduction of more than 10 dB. By elucidating both macroscopic equivalent parameters and microscopic power losses, this work provides a practical pathway for satellite electromagnetic purification and radar stealth.

## 2. Methods and Results

### 2.1. Solar Panel Substrate and Simulation Substitution Basis

During the execution of space missions, the startup and operation of satellite payloads consume large amounts of electrical energy. Therefore, autonomous energy supply capability is a necessary condition for ensuring normal operation. According to relevant statistics, about 90% of satellites currently in orbit use solar energy as the main power generation method, making solar panel the core component of satellite energy supply. Their operating mechanism is based on the photoelectric conversion principle. By receiving solar radiation, they convert light energy into electrical energy and store it via batteries, thereby providing stable power support for spacecraft such as satellites and spaceships.

**Figure 1:**
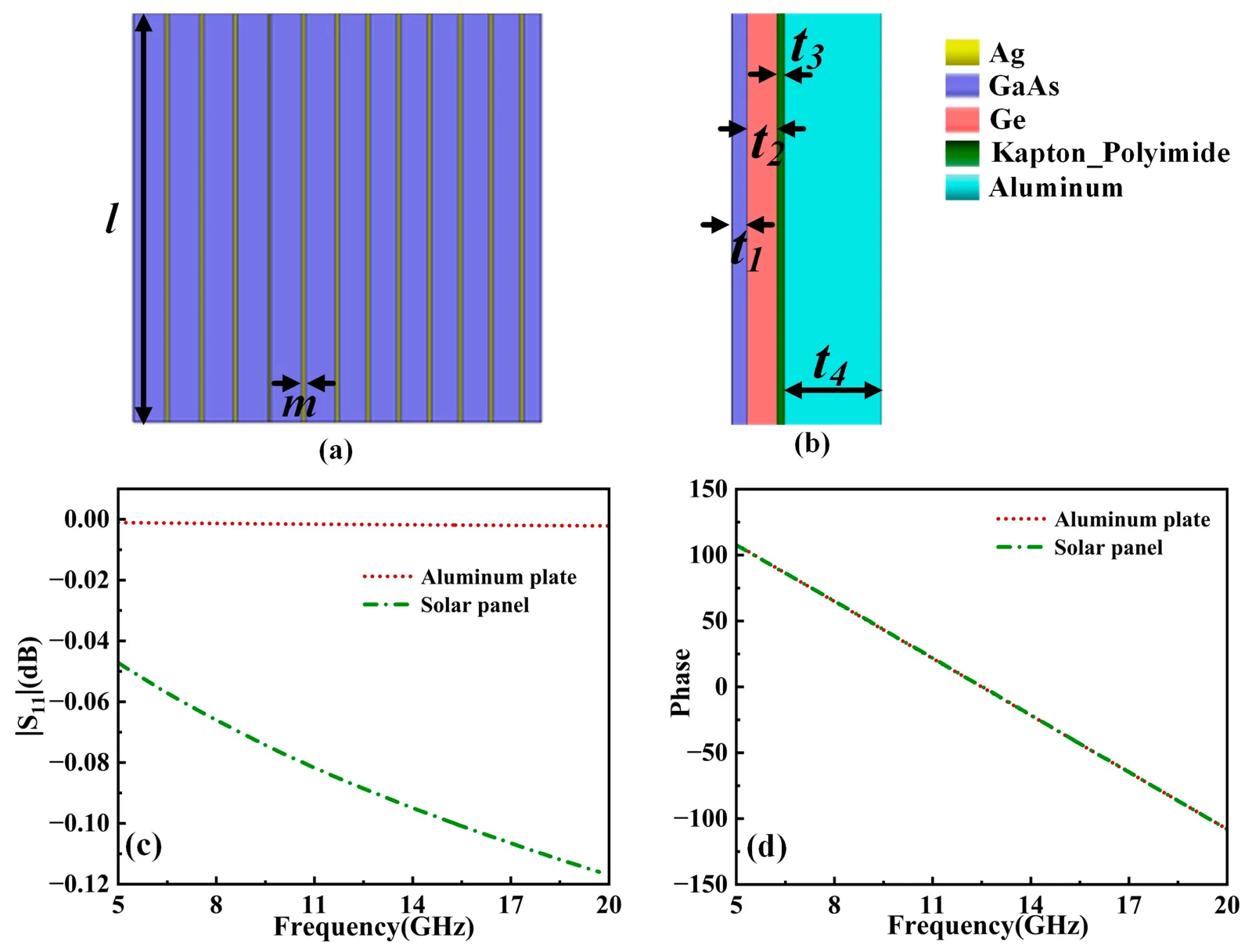
(**a**) Top view of the solar panel model (lateral dimension). (**b**) Side view of the multi-layer structure. Comparison of the simulated (**c**) reflection amplitude and (**d**) phase between the solar panel and an aluminum plate.

To verify the metal-like reflection characteristics of the solar panel, a realistic multi-layer model was constructed in CST Microwave Studio. Figure 1a,b illustrate the detailed geometry with a lateral dimension of *l* = 12 mm. From top to bottom, the structure consists of a GaAs triple-junction active cell (t_1_ = 0.1 mm), a Ge substrate (t_2_ = 0.3 mm), a Kapton polyimide film (t_3_ = 0.05 mm), and an aluminum honeycomb core (t_4_ = 0.65 mm). On the top surface, a Ag grid is printed directly onto the GaAs layer, occupying a metal coverage ratio of 8% (m = 0.08 mm). This closely matches the actual metal fraction of a real solar panel. For the electromagnetic simulation, the semiconductor components (GaAs and Ge) were treated as lossy conductors with relative permittivity of 13 and 16, and high conductivities of 10,000 S/m and 20,000 S/m, respectively, while their dielectric loss tangents were neglected. In contrast, the Kapton film (ε = 3.2, tan δ = 0.003) and the aluminum honeycomb core (ε = 1.15, tan δ = 0.001) were modeled as low-loss dielectrics.

For the full-wave simulation using the FEM solver in CST, periodic boundary conditions (unit cell) were applied along the x- and y-axes to mimic an infinite array, while an open add space boundary was set along the *z*-axis. Electromagnetic waves were normally incident from port Z_max_ to Z_min_, maintaining a far-field distance. Figure 1c,d compare the simulated reflection properties between this realistic model and an ideal aluminum plate. In Figure 1c, the reflection amplitude ∣S_11_∣ of the aluminum plate remains steadily at 0 dB, whereas the solar panel fluctuates only marginally between −0.04 and −0.12 dB across 5–20 GHz. This negligible amplitude difference indicates that, despite the complex dielectric stacks and Ag grid, the solar panel behaves macroscopically as a strong reflector. Furthermore, Figure 1d reveals that the reflection phase of the solar panel almost perfectly overlaps with that of the aluminum plate.

**Figure 2:**
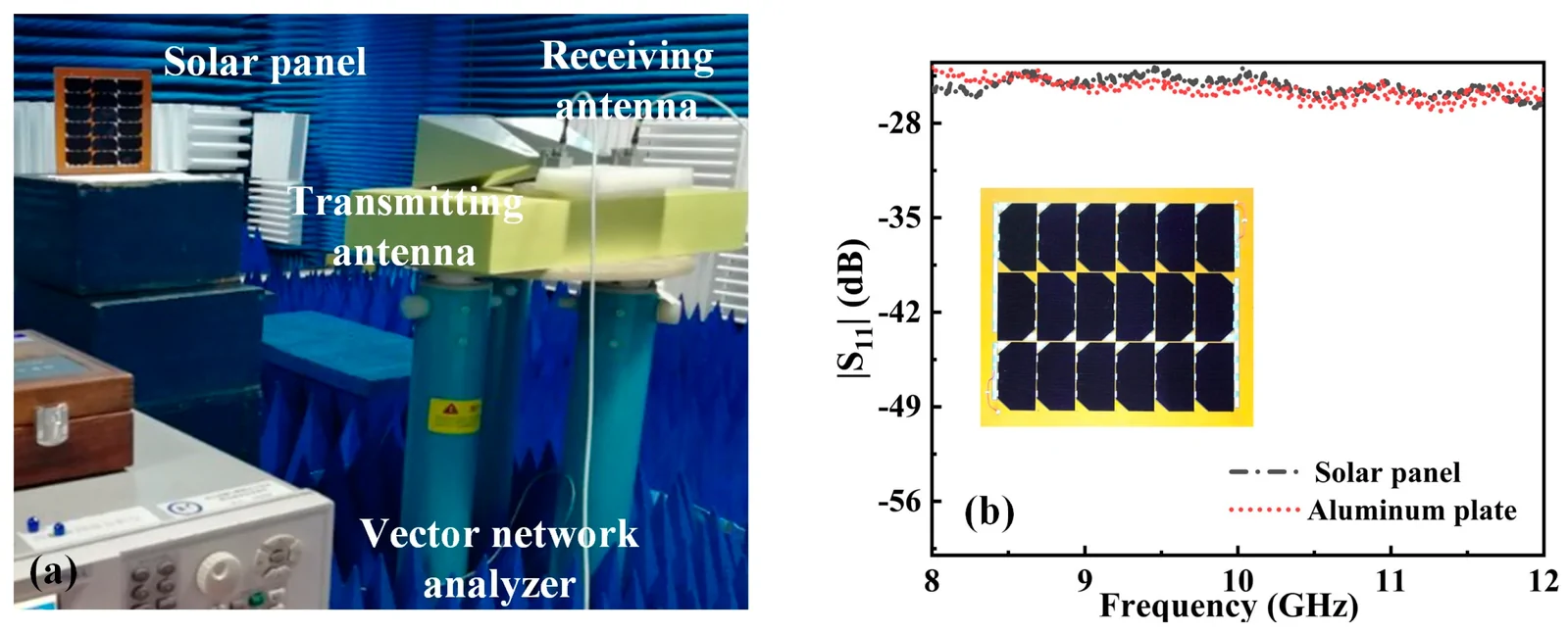
Experimental verification of the metal-like properties: (**a**) Free-space measurement setup in a microwave anechoic chamber; (**b**) Measured reflection coefficients (∣S_11_∣) of the solar panel and aluminum plate.

Complementing the numerical analysis, a free-space measurement was conducted in a microwave anechoic chamber (Figure 2a). The measured reflection coefficients ∣S_11_∣ in the 8-12 GHz band (Figure 2b) show that the solar panel aligns almost perfectly with the aluminum plate, with both fluctuating closely around −28 dB.

Both simulated and measured data strongly confirm the robust metal-like characteristics of the solar panel. Its reflection behavior—in both amplitude and phase—is practically indistinguishable from an ideal metal in the microwave band. Consequently, substituting the solar panel with an aluminum plate in subsequent full-wave simulations and experimental evaluations is highly justified.

**Figure 3:**
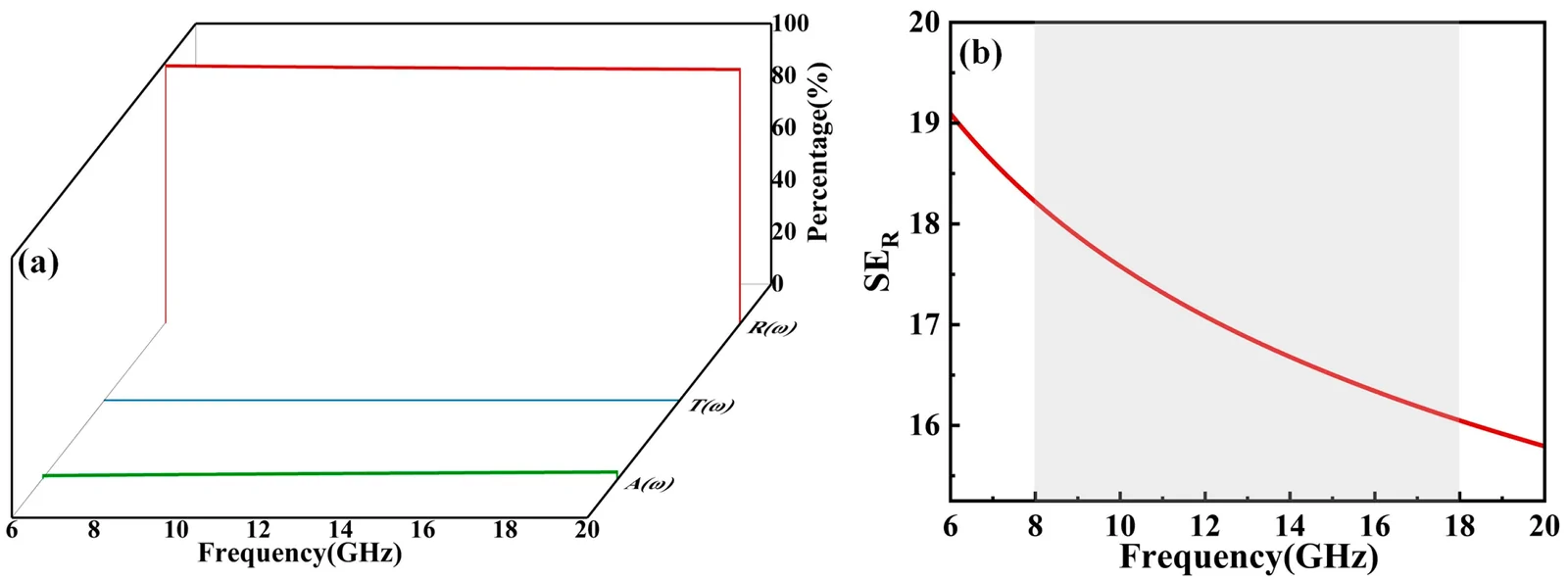
Solar panel: (**a**) reflectance, transmittance, and absorptivity; (**b**) reflection shielding effectiveness.

To quantify the initial shielding state of the bare solar panel, the simulated reflection, transmission, and absorption rates, along with the calculated reflection shielding effectiveness (SE_R_), are presented in Figure 3. As shown in Figure 3a, the solar panel exhibits near-total reflection across the 6–20 GHz band, with R(ω) hovering around 99% and A(ω) remaining close to zero. Due to the presence of the opaque aluminum honeycomb core and the Ge substrate, the transmission rate T(ω) is approximately zero. Based on the reflection data, the corresponding SE_R_ is calculated and plotted in Figure 3b. The SE_R_ value varies between 15.8 dB and 19.1 dB in this frequency range. Since this value is substantially higher than the 3.01 dB criterion, it definitively confirms that the bare solar panel is a classic reflection-dominated shielding material, where the incident electromagnetic energy is primarily redirected rather than dissipated.

### 2.2. Unit Structure

**Figure 4:**
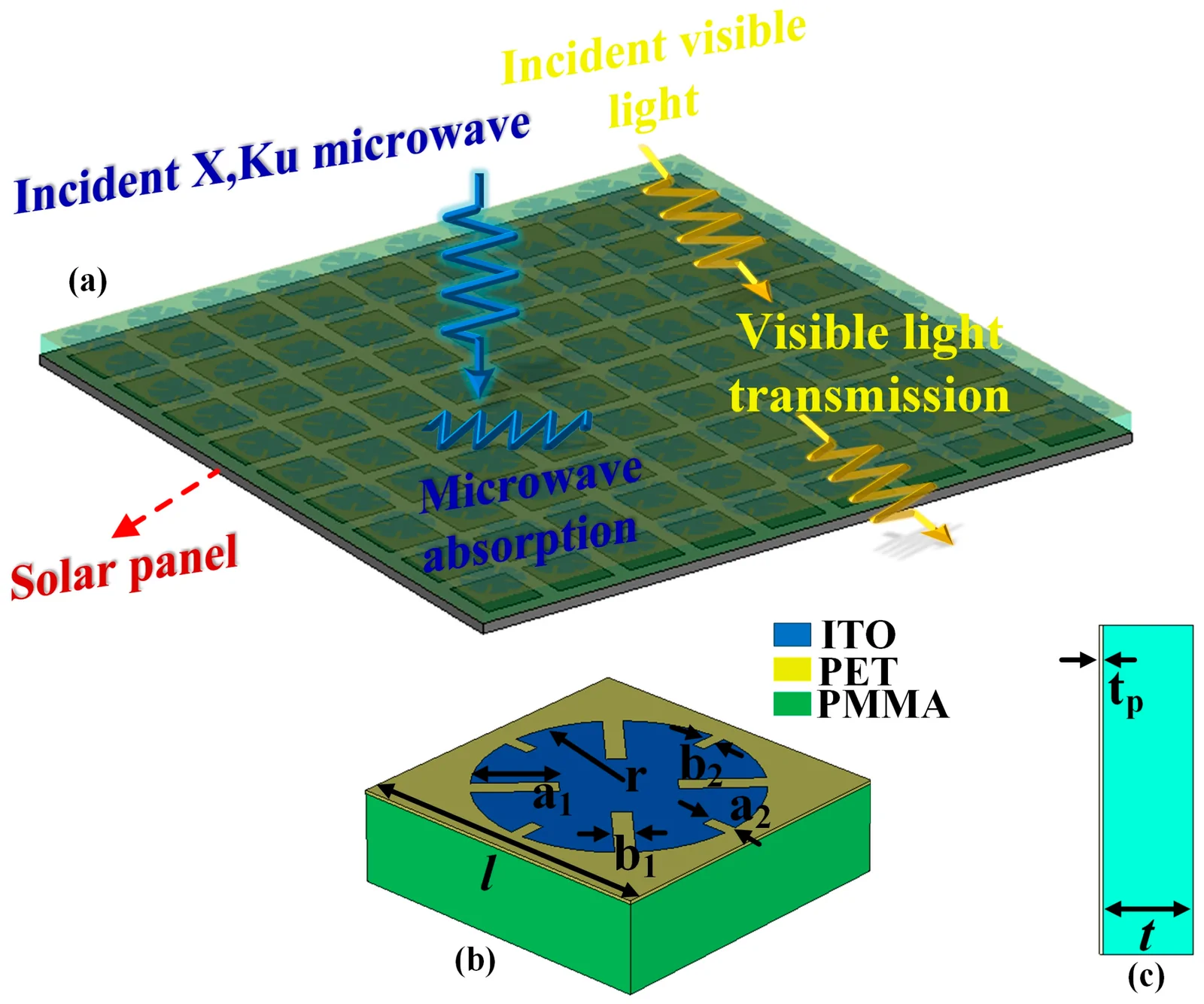
(**a**) Conceptual diagram of the OTMMA system; (**b**) 3D view of the unit structure; (**c**) side view of the unit structure.

To convert the shielding mechanism of the solar panel, we constructed an optically transparent metamaterial absorber (OTMMA) using the solar panel as the substrate. Its working concept is shown in Figure 4a. When electromagnetic waves in the X- and Ku-bands are incident on the OTMMA, good impedance matching at the OTMMA surface allows the waves to enter with almost no reflection. The electromagnetic waves entering the OTMMA are absorbed due to ohmic loss, and the solar panel exhibits an absorption-type shielding mechanism. Due to the selection of transparent materials, visible light incident on the OTMMA surface can pass through the structure and reach the underlying substrate, as confirmed by the optical transmittance measurements.

The unit cell, shown in Figure 4b,c, comprises a patterned ITO resistive layer on a 0.125 mm PET substrate, a PMMA dielectric spacer, and the solar panel itself as the reflective ground. The ITO layer was deposited by magnetron sputtering and etched into a circular pattern with a sheet resistance of 60 ohm/sq. The PMMA and PET parameters are ε_PMMA_ = 2.25, tan δ_PMMA_ = 0.001, and ε_PET_ = 3, tan δ_PET_ = 0.06, respectively. We used CST for full-wave simulation with the same boundary conditions as above. The optimized structural parameters are: *l* = 12.0 mm, r = 5.0 mm, a_1_ = 2.99 mm, a_2_ = 0.99 mm, b_1_ = 0.7 mm, b_2_ = 0.5 mm, t = 3.3 mm, t_p_ = 0.125 mm.

### 2.3. S-Parameters and Absorptivity

**Figure 5:**
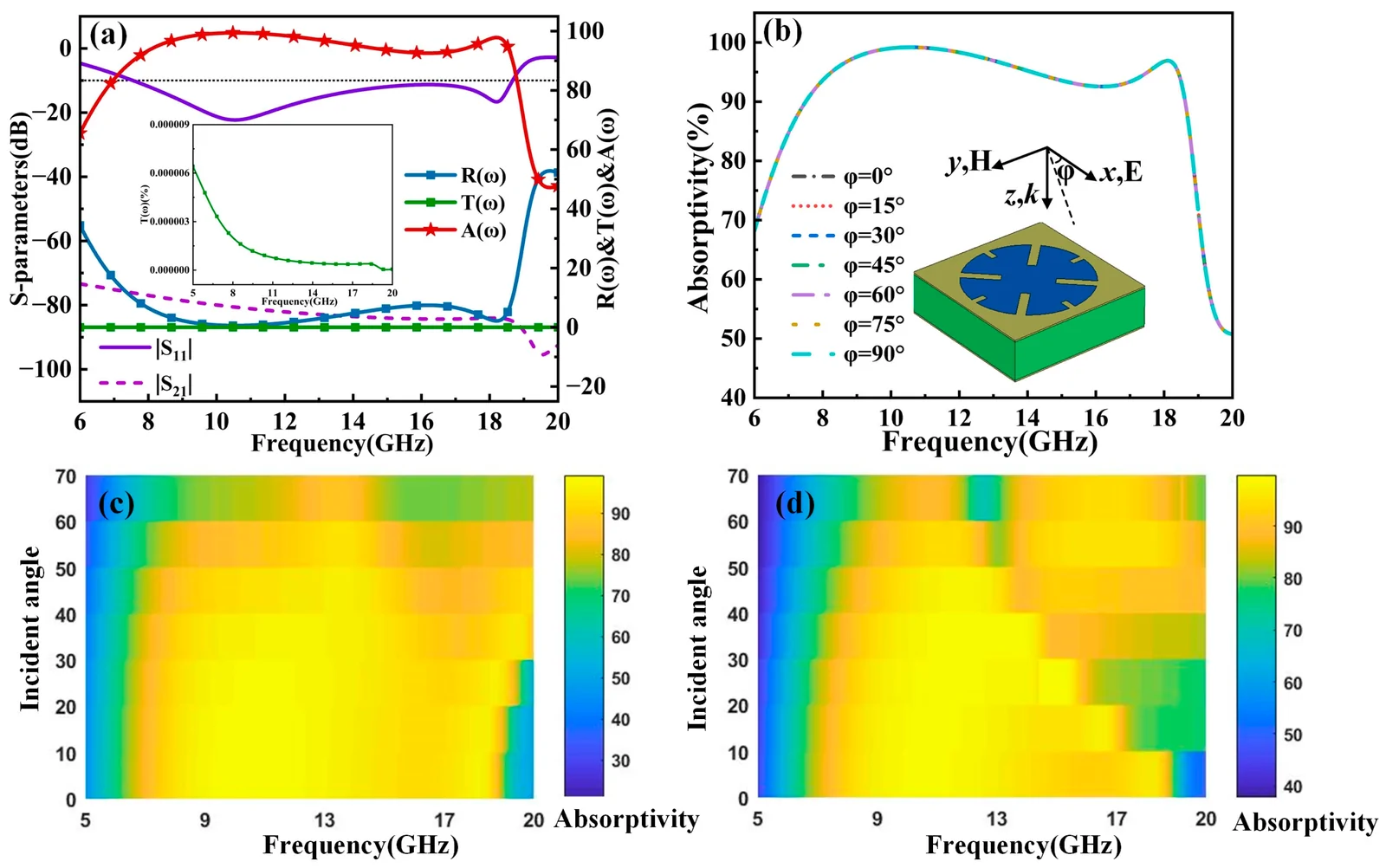
OTMMA: (**a**) S-parameters, R(ω), T(ω), and A(ω); (**b**) A(ω) at different polarization angles and A(ω) at different incidence angles; (**c**) TE polarization; (**d**) TM polarization.

Under normal incidence, the simulated S-parameters along with the reflectance, transmittance, and absorptance curves of the OTMMA are shown in Figure 5a. The reflection coefficient ∣S_11_∣ remains below −10 dB from 7.63 to 18.71 GHz, indicating excellent impedance matching at the OTMMA surface. Because the solar panel serve as the substrate, wave propagation is heavily blocked. As clearly shown in the inset of Figure 5a, the transmittance T(ω) remains nearly zero across the entire frequency range. For normal incidence, the absorptivity A(ω) is evaluated using the complete relation A(ω) = 1 − R(ω) − T(ω), where the reflectance and transmittance are extracted directly from the simulated S-parameters as R(ω) = ∣S_11_∣^2^ and T(ω) = ∣S_21_∣^2^. Benefiting from the strong impedance matching and near-zero transmission, the resulting absorptivity (red dashed line) exceeds 90% across the entire 7.63–18.71 GHz band. Notably, a distinct absorption peak approaching 100% is achieved at 10.68 GHz. This outstanding broadband absorption performance covers both the X-band (8–12 GHz) and Ku-band (12–18 GHz), fully verifying the effectiveness of the structural design.

The polarization insensitivity of the design is verified in Figure 5b. As the polarization angle φ varies from 0° (TE) to 90° (TM), the absorptance curves practically overlap across the entire frequency range. This behavior is attributed to the fourfold rotational symmetry of the unit cell geometry (as illustrated in the inset). Furthermore, the evolution of the >90% absorption bandwidth with respect to the incidence angle is analyzed in Figure 5c,d, where the yellow regions represent absorptance exceeding 90%. Under TE polarization (Figure 5c), the full design band of >90% absorption is remarkably stable up to an incidence angle of 50°. As the angle increases to 60°, the bandwidth slightly narrows to approximately 8.5–18.0 GHz. At 70°, high-frequency degradation becomes evident, leaving a narrower >90% band of roughly 9.5–16.0 GHz. For TM polarization (Figure 5d), the angular stability is even more robust. The full >90% absorption band is well-maintained up to 60°. Even at a steep angle of 70°, the structure still sustains a broad >90% absorption from approximately 7.0 to 17.0 GHz. These results collectively demonstrate that the proposed OTMMA possesses excellent wide-angle stability while maintaining high absorption.

**Figure 6:**
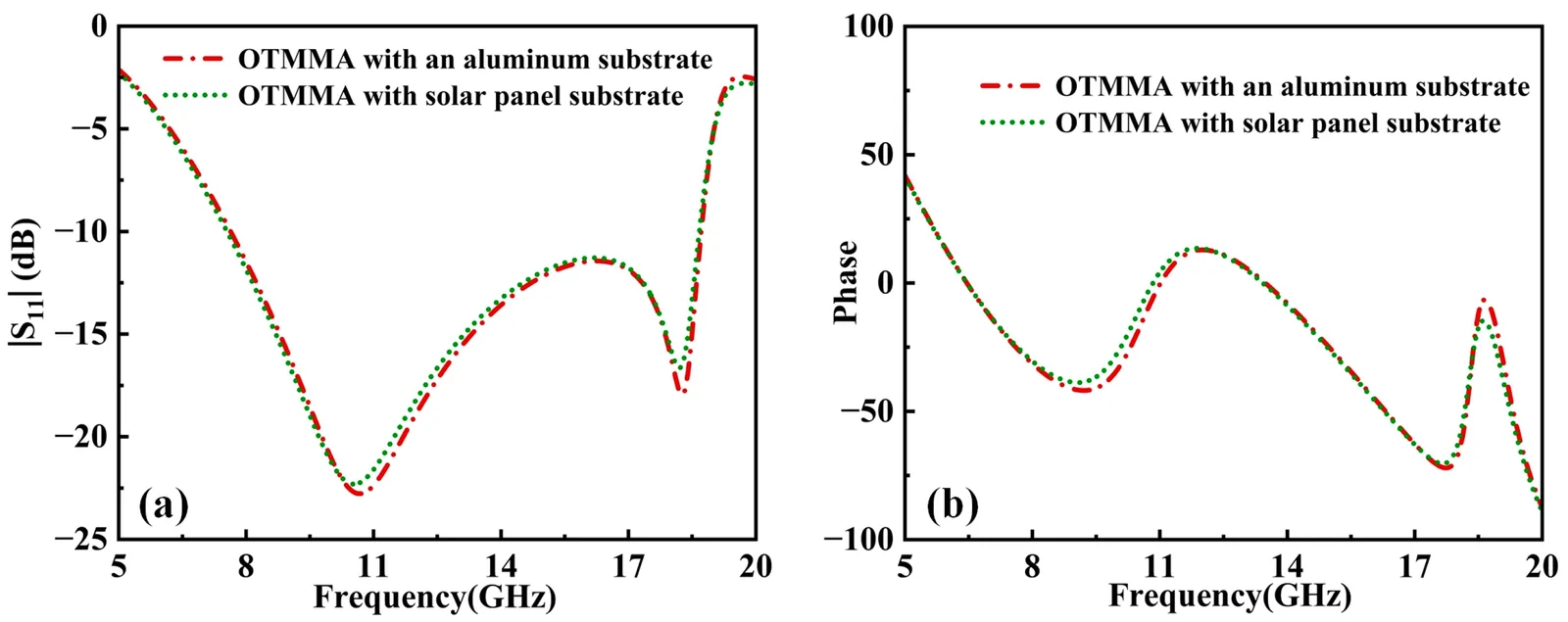
Comparison of the complete OTMMA response using an aluminum substrate and a realistic solar panel substrate: (**a**) Reflection amplitude (∣S_11_∣); (**b**) Reflection phase.

To further validate that this substrate substitution does not alter the resonant behavior of the complete absorber, the full OTMMA was simulated on both an aluminum substrate and the realistic solar panel substrate. As shown in Figure 6, the reflection amplitude (∣S_11_∣) and phase of the complete OTMMA on both substrates are virtually indistinguishable, exhibiting the identical absorption resonance at 10.7 GHz. This confirms that substituting the solar panel with an aluminum plate is highly reliable for evaluating the complete absorber performance.

### 2.4. Shielding Mechanism Conversion Analysis

According to the different ways of blocking incident electromagnetic waves, shielding materials can be divided into reflection-type and absorption-type. Reflection-type shielding materials cannot eliminate incident electromagnetic waves but only redirect them, causing additional electromagnetic pollution and interference. In contrast, absorption-dominated shielding materials can exclusively eliminate incident or self-induced electromagnetic waves. The criterion for absorption-type shielding materials is that the reflection shielding effectiveness SE_R_ is less than 3.01 dB [24]. When SE_R_ < 3.01 dB, the material’s reflection contribution is extremely low, and the shielding mechanism is absorption-dominated.

For a metal-backed metamaterial absorber, this SE_R_ criterion mathematically corresponds to a reflectance R < 50%. While conventional metamaterial absorbers typically prioritize radar stealth (reducing backward echoes) and tolerate certain reflection levels, satellite systems demand stricter electromagnetic compatibility (EMC). A bare solar panel acts as a highly reflective pollution source (SE_R_ > 15 dB). By reducing its SE_R_ below 3.01 dB, the surface boundary condition is fundamentally altered: the panel no longer merely reflects waves, but becomes an absorption-dominant surface that prevents internal crosstalk among onboard electronics.

**Figure 7:**
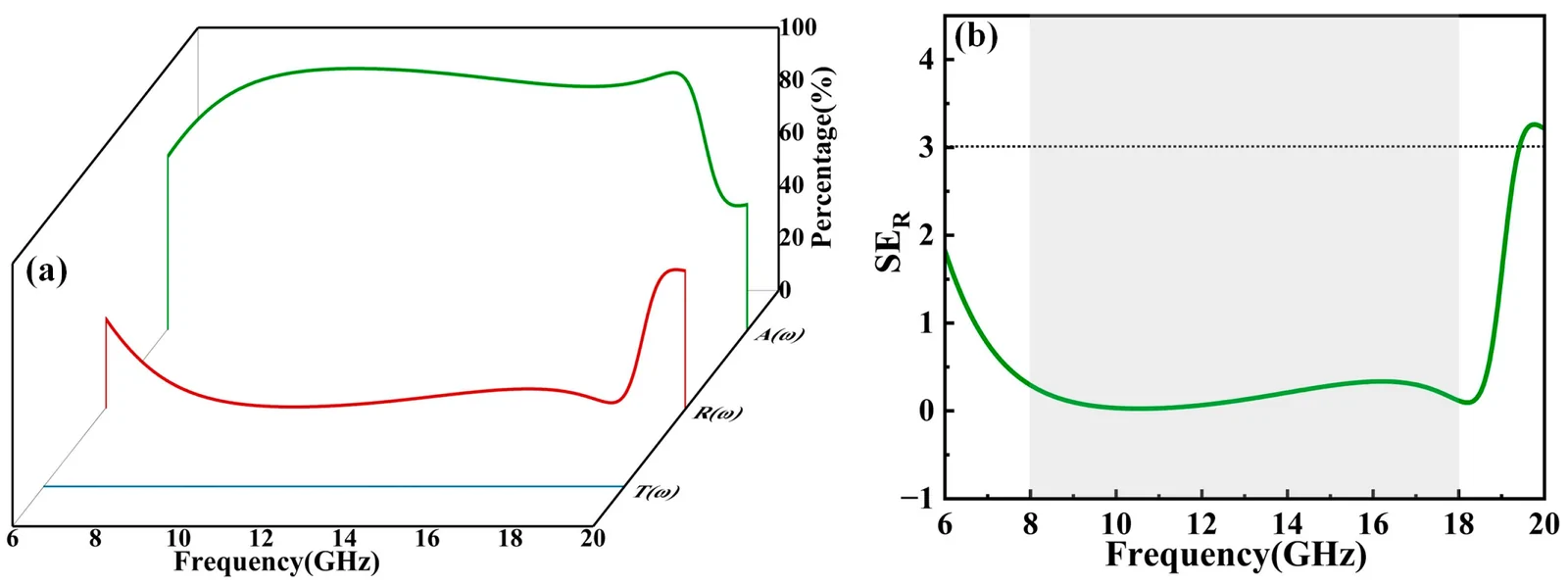
(**a**) Reflectance, transmittance, and absorptivity of the covered solar panel; (**b**) reflection shielding effectiveness.

To evaluate this conversion, the SE_R_ is calculated as SE_R_ = −10lg|1 − R|. Using the simulated S-parameters, the reflectance, transmittance, and absorptance are derived in Figure 7a. Compared to the bare panel, the OTMMA-covered panel achieves a transition from high reflection to over 90% absorption. As shown in Figure 7b, the SE_R_ drops significantly from over 15 dB (bare panel) to less than 3.01 dB across the entire design band (shaded area). This confirms the successful conversion of the solar panel from a reflective EMI source into an absorption-dominant purifying surface.

### 2.5. Radar Cross Section (RCS) Reduction Analysis

Beyond absorptance, the radar stealth performance was evaluated via CST full-wave simulations. To specifically evaluate the backscattering signature, the monostatic RCS was simulated for a finite 300 × 300 mm^2^ plate under normal-incidence, x-polarized plane wave conditions. To ease the computational burden while maintaining accuracy, the solar panel was substituted with an aluminum plate, as validated in Section 2.1 and Section 2.3. The x, y, and z-directions were set as open boundaries, and an x-polarized plane wave was applied to calculate the far-field monostatic RCS.

**Figure 8:**
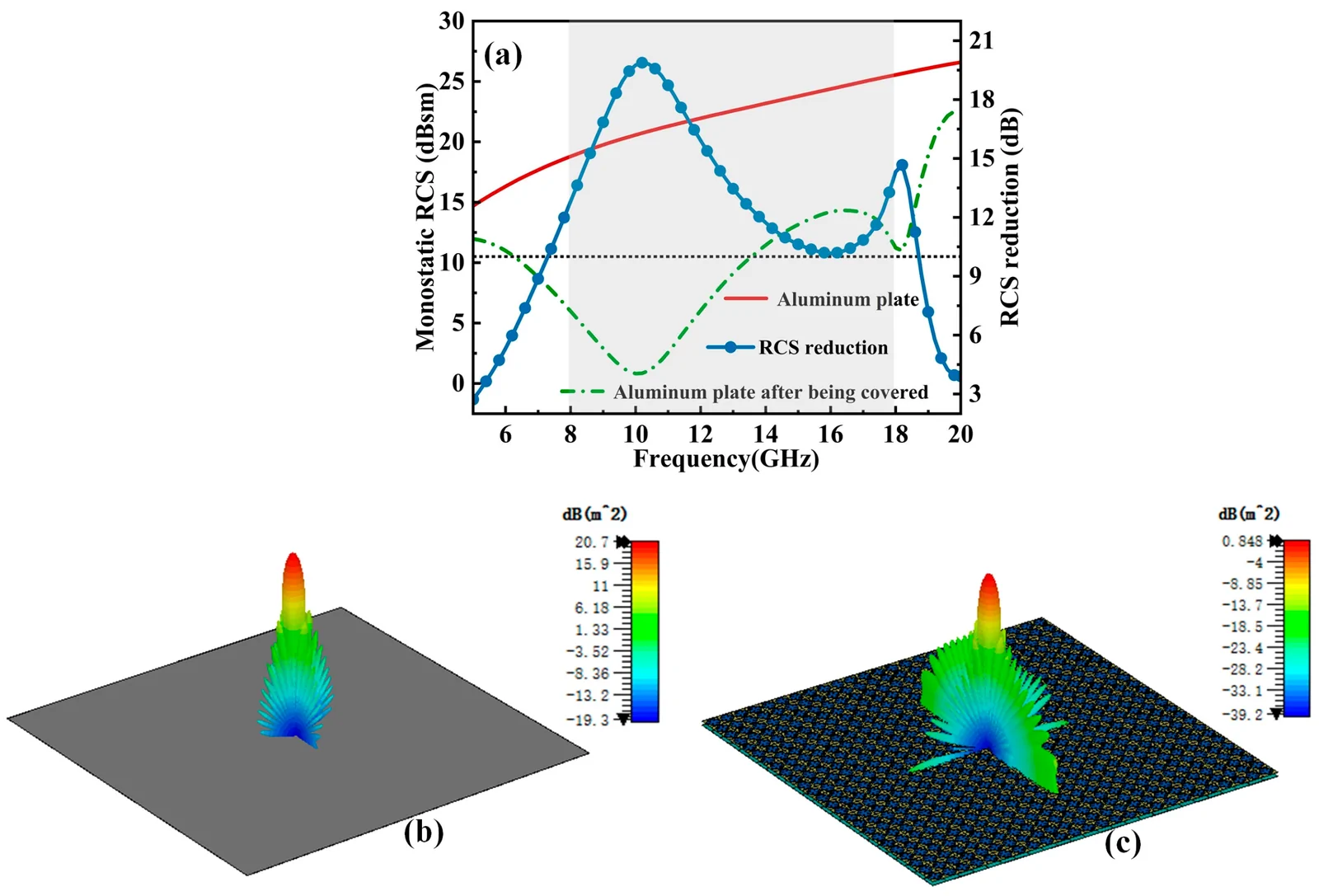
RCS simulation results: (**a**) Monostatic RCS and the corresponding RCS reduction in the bare plate and the OTMMA-covered plate; 3D bistatic RCS patterns at 10.7 GHz for (**b**) the bare plate and (**c**) the OTMMA-covered plate.

Figure 8a contrasts the monostatic RCS of the bare plate and the OTMMA-covered plate. The bare plate exhibits a strong scattering signature, with its RCS values fluctuating above 10 dBsm (red curve). Once covered by the absorber, the RCS (blue curve) experiences a dramatic drop. A consistent reduction of more than 10 dB is achieved across the entire 6–20 GHz band, and this suppression peaks at 10.7 GHz. The 3D bistatic RCS patterns at 10.7 GHz visually illustrates this dramatic suppression. For the bare plate (Figure 8b), a prominent main lobe points directly backward, indicating severe reflection. In contrast, the covered plate (Figure 8c) almost completely scatters this backward lobe, forcing the residual energy to spread outward. At this specific frequency, the peak backward RCS drops drastically, yielding a maximum reduction of 20.43 dB. These simulations demonstrate that, for a finite plate under normal incidence, the proposed OTMMA can significantly suppress backward scattering, thereby reducing the radar detection signature compared to an uncoated panel.

## 3. Analysis and Discussion

### 3.1. Equivalent Parameter Retrieval

To investigate the mechanisms by which the solar panel achieves electromagnetic purification and radar stealth, the subwavelength OTMMA was modeled as a homogeneous slab with an effective thickness *t*. The equivalent parameters were extracted based on effective medium theory. Based on the transfer matrix method, the equivalent refractive index n and relative impedance z can be analytically derived from the simulated S-parameters (S_11_ and S_21_) as:

(1) $$n=\frac{1}{\mathrm{kt}}\cos^{-1}\left[\frac{1}{2S_{21}}(1-S_{11}^{2}+S_{21}^{2})\right]$$

(2) $$z=\sqrt{\frac{\left(1+S_{11}^{2}\right)-S_{21}^{2}}{\left(1-S_{11}^{2}\right)-S_{21}^{2}}}$$

where *k* is the free-space wavenumber. The effective relative permittivity and permeability are then calculated as ε = *n*/*z* and μ = *n*⋅*z*.

Because the structure is ground-backed by an opaque conductive layer, the transmission coefficient S_21_ is extremely small. In Formula (1), dividing by this near-zero S_21_ causes the inverse cosine function to become highly sensitive to numerical noise and mathematically multi-valued. If left unaddressed, this instability leads to severe non-physical fluctuations and spurious values in the retrieved ε and μ. To resolve this and ensure physical validity, a branch-selection procedure was strictly enforced based on the passivity condition (Im (*n*) ≥ 0). Consequently, the retrieved negative real parts of ε and μ are confirmed to represent the intrinsic macroscopic resonant response of the metamaterial, rather than being numerical artifacts of the near-zero S_21_.

**Figure 9:**
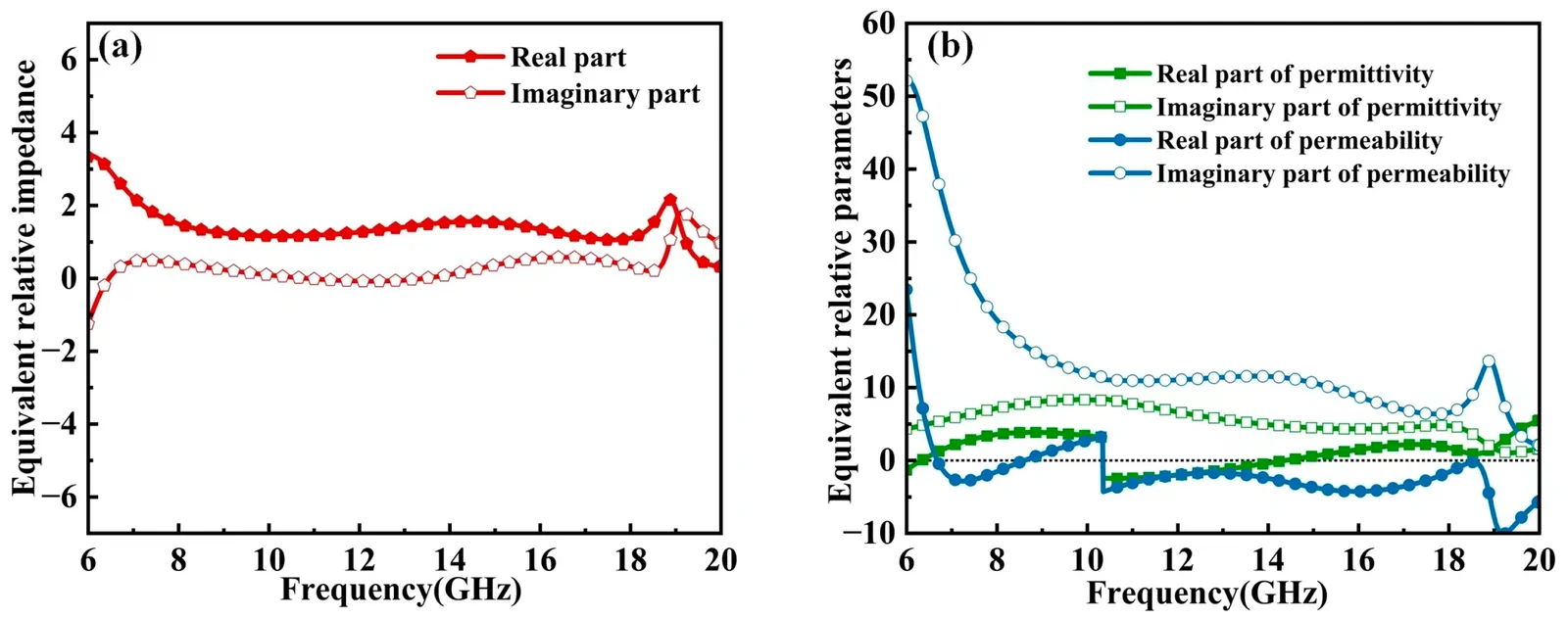
OTMMA: (**a**) equivalent impedance; (**b**) equivalent permittivity and permeability.

As shown in Figure 9a, in the 8–18 GHz range, the real part of the equivalent relative impedance fluctuates around 1, and the imaginary part fluctuates around 0. This means that when incident electromagnetic waves reach the OTMMA surface, the equivalent relative impedance achieves good matching with free space, allowing the waves to penetrate into the material with nearly zero reflection. Figure 9b shows the retrieved permittivity and permeability. In the design band, the real parts of both parameters become less than 0, indicating typical metamaterial electromagnetic response characteristics. The imaginary parts are both greater than 0, proving that the OTMMA has dielectric loss and magnetic loss. The good matching between the equivalent impedance and free space ensures low-reflection entry of incident waves, while the positive imaginary parts ensure effective loss of electromagnetic waves after they enter the interior. Together, these two factors correspond to the absorptance above 90% in Section 2.3.

These equivalent parameters describe the overall macroscopic response of the OTMMA, yet the loss mechanisms indicated by their imaginary parts need to be traced at the microscopic level. Accordingly, the subsequent sections will reveal the resonance excitation mechanisms via field distributions (Section 3.2) and analyze the spatial power loss density (Section 3.3).

### 3.2. Field Distribution

While Section 3.1 treated the OTMMA as a macroscopic medium exhibiting equivalent dielectric and magnetic losses, this section breaks down the structure to reveal the microscopic physical origin of these losses.

**Figure 10:**
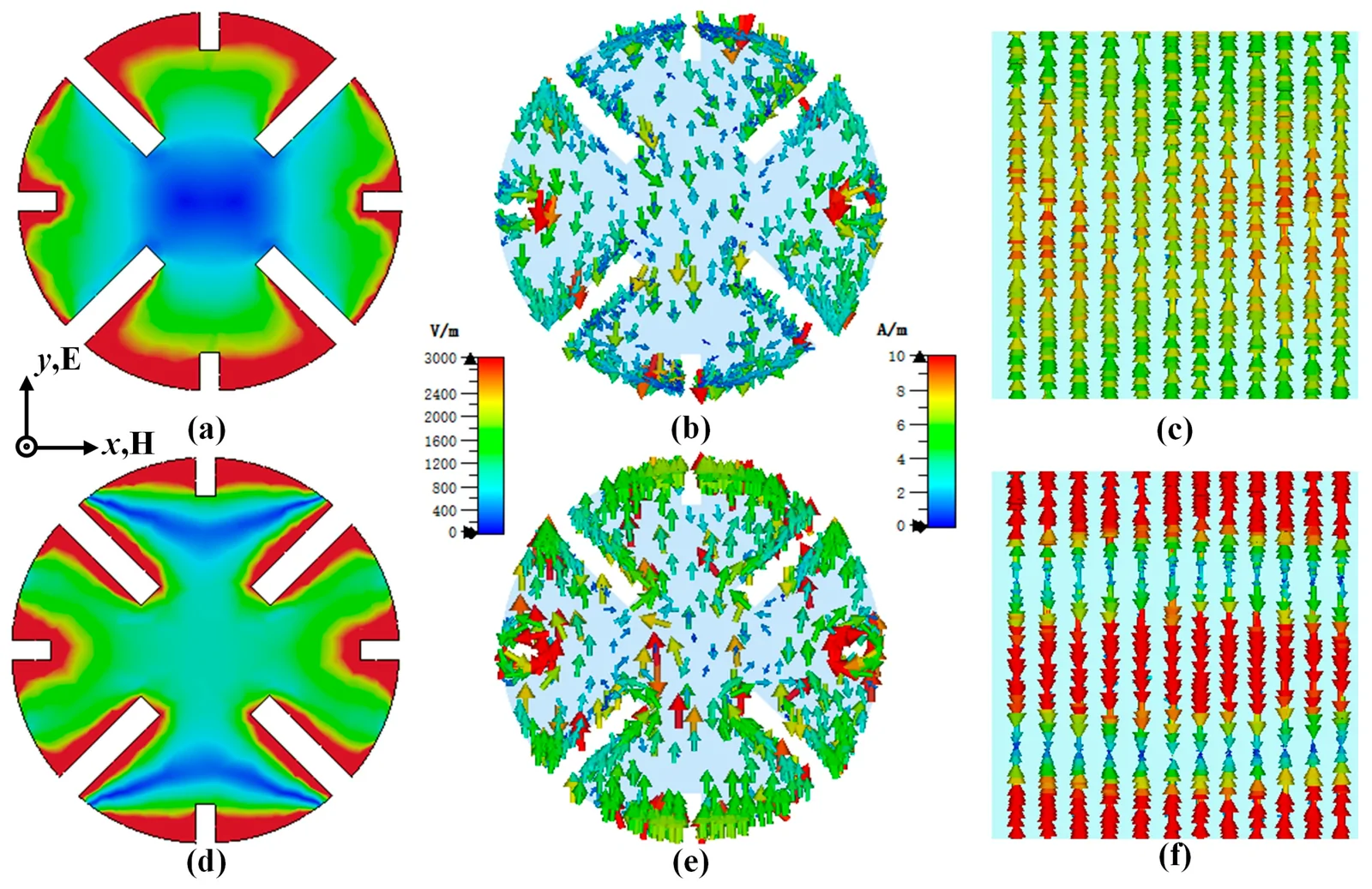
At 10.68 GHz: (**a**) electric field distribution of the top ITO layer; (**b**) surface current distribution; (**c**) surface current distribution of the Ag grid substrate. At 18.27 GHz: (**d**) electric field distribution of the top ITO layer; (**e**) surface current distribution; (**f**) surface current distribution of the Ag grid substrate.

Figure 10 shows the surface electric field and current distributions at the resonance points of 10.68 and 18.27 GHz under normal incidence and TE polarization. As shown in Figure 10a,d, the electric field of the top ITO layer is mainly concentrated in the opening direction at an angle to the *y*-axis. The incident electric field is along the *y*-axis. When charges move along the *y*-axis, the opening at an angle to the *y*-axis acts as a capacitor, and a large amount of charge accumulates along the *y*-axis. The large accumulation of charge indicates the generation of electric resonance. For the opening parallel to the electric field direction, the electric field is mainly distributed on both sides of the opening, originating from the capacitance formed by the gaps between units. Figure 10b,e show the current distribution of the top ITO layer, and Figure 10c,f show the current distribution of the Ag grid at the two resonance points. At each resonance point, oppositely flowing currents exist in the top layer and the substrate, forming a circulating loop current in the entire OTMMA. Under the action of this circulating current, magnetic resonance is excited. After electromagnetic resonance is excited, the OTMMA allows more of the incident electromagnetic waves to enter the interior. Because ohmic loss exists, the electromagnetic energy incident into the interior is gradually absorbed, ultimately achieving high absorption of the solar panel.

At the two resonance points, electric resonance and magnetic resonance are simultaneously excited. Electric resonance is mainly generated by charge accumulation at the openings of the top ITO layer, and magnetic resonance is mainly generated by the reverse circulating current between the top ITO and the substrate. The combined action enables the OTMMA to achieve near-100% absorption at these two frequency points. Between the two resonance points, the intensities of electric and magnetic resonance decrease somewhat, but sufficient electromagnetic energy still enters the OTMMA interior and is absorbed by ohmic loss, thereby maintaining broadband absorption above 90% in the 7.63–18.71 GHz range.

It should be noted that electric resonance and magnetic resonance are not independently generated by “two layers of materials.” Electric resonance mainly originates from charge accumulation of the top ITO pattern under the incident electric field, while magnetic resonance originates from the equivalent current loop formed by the reverse flowing surface currents between the top ITO and the substrate metal. The role of the substrate metal is to provide a reflective surface and form a current loop with the top layer, rather than independently exciting magnetic resonance. Their synergistic action enables the OTMMA to achieve impedance matching at the resonance frequencies, thereby allowing electromagnetic waves to enter the structure interior with low reflection.

### 3.3. Surface Power Loss Density

**Figure 11:**
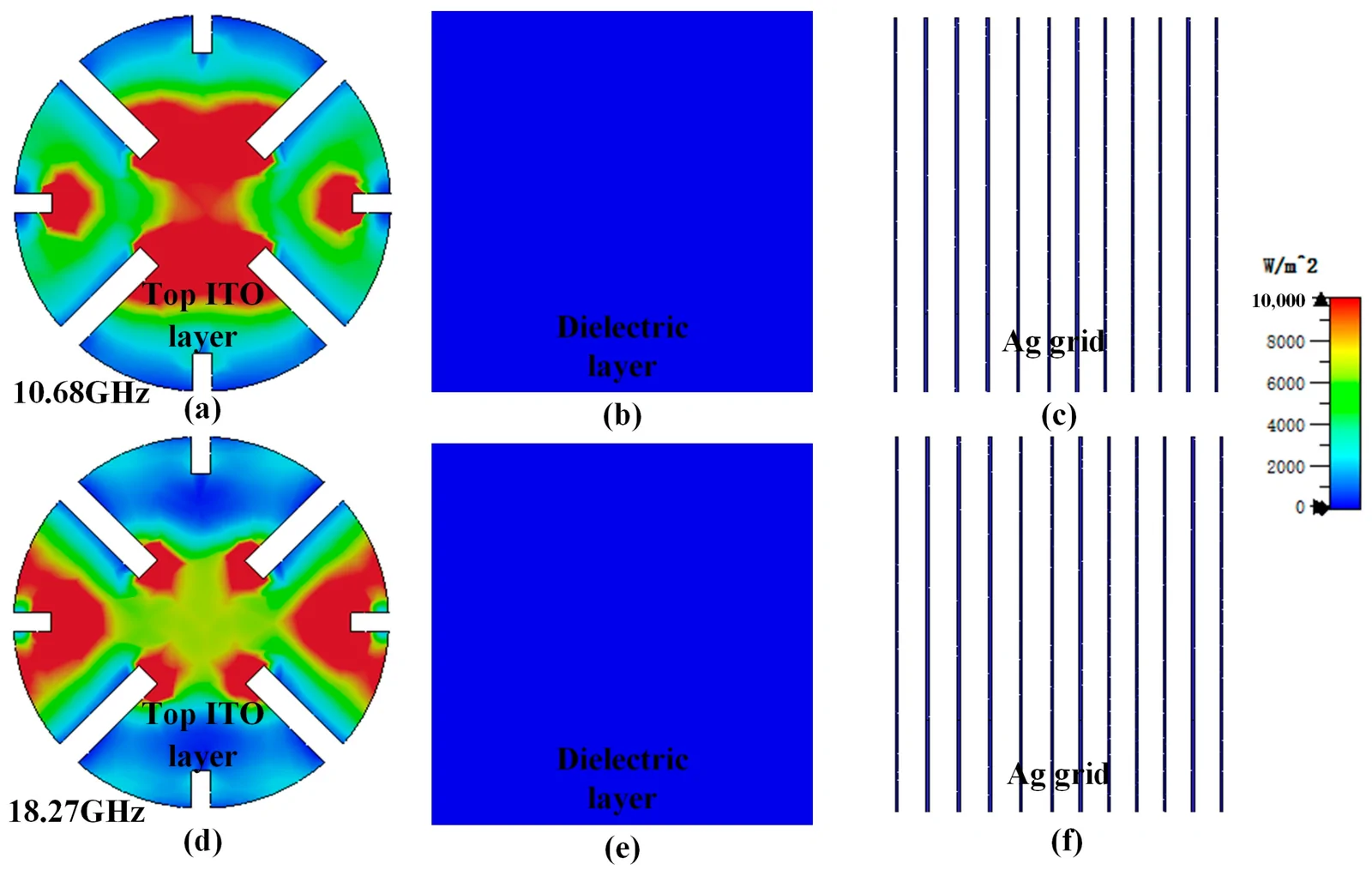
Surface power loss density at 10.68 GHz: (**a**) top ITO layer; (**b**) dielectric layer; (**c**) Ag grid. Surface power loss density at 18.27 GHz: (**d**) top ITO layer; (**e**) dielectric layer; (**f**) Ag grid.

The previous two sections explained the impedance matching and resonance mechanisms from the perspectives of equivalent parameters and field distribution. To further locate the specific source of incident electromagnetic wave loss, we simulated the surface power loss densities at 10.68 GHz and 18.27 GHz, as shown in Figure 11a–f. Taking 10.68 GHz as an example, from left to right are the top ITO layer, dielectric layer and Ag grid. Note that the dielectric layer column collectively represents the PMMA, PET, and the dielectric components within the solar panel. The arrangement at 18.27 GHz is the same. Power loss is mainly concentrated in the top ITO layer, and the top ITO layer mainly exhibits ohmic loss. At 10.68 GHz, the order of surface power loss density is (a) > (b) = (c). At 18.27 GHz, the order is (d) > (e) = (f), where (a), (c), (d), and (f) are ohmic losses, and (b) and (e) are dielectric losses.

The ITO pattern layer serves a dual function in the OTMMA. On one hand, it regulates the overall equivalent impedance through the pattern geometry to match free space and reduce surface reflection. On the other hand, the surface currents excited on the ITO pattern after electromagnetic waves enter generate ohmic loss due to the finite conductivity of ITO. Since the dielectric loss tangent of dielectric layer is extremely small and Ag grid has no ohmic loss, the energy dissipation in the OTMMA almost entirely occurs in the ITO layer. This is consistent with the field distribution in Figure 10, where the current density on the ITO pattern is visibly higher than on the substrate. The resonance mechanisms ensure that electromagnetic waves can enter the structure interior, while the ohmic loss of ITO converts these entered electromagnetic energies into heat. The high absorption of the solar panel therefore mainly relies on the ohmic loss of the top ITO layer. It is worth noting that while Section 3.1 treated the OTMMA as a homogeneous macroscopic medium exhibiting both equivalent dielectric and magnetic losses, breaking the structure down here reveals the microscopic origin of these losses. In reality, the macroscopic dielectric and magnetic losses are primarily manifested as the microscopic ohmic loss of the ITO layer. Ultimately, while the resonance mechanisms facilitate the entry of electromagnetic waves, the ohmic dissipation of the ITO pattern converts this trapped energy into heat, driving the broadband high absorption of the solar panel.

## 4. Experimental Verification

### 4.1. S-Parameter and Absorptivity Measurements

**Figure 12:**
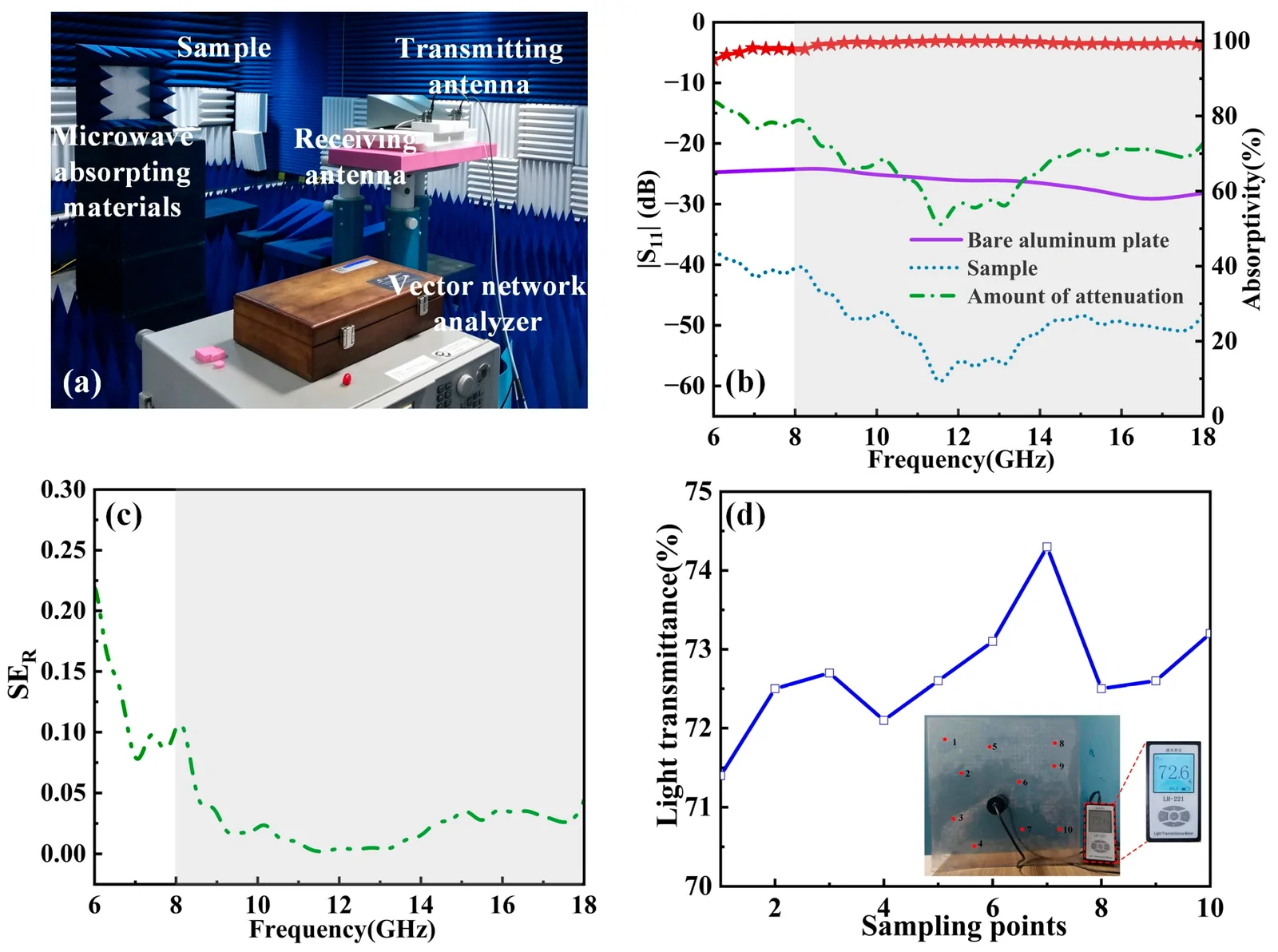
Experimental results: (**a**) Free-space measurement setup in the anechoic chamber; (**b**) measured reflection coefficient ∣S_11_∣ of the sample and bare aluminum plate, along with the calculated attenuation and absorptance; (**c**) measured reflection shielding effectiveness; (**d**) measured optical transmittance at different sampling points.

The microwave performance was evaluated in a fully anechoic chamber using the free-space method. As shown in Figure 12a, standard broadband horn antennas were connected to Port 1 (Tx) and Port 2 (Rx) of the VNA and placed on the same side of the sample at a distance of 2.0 m. This distance satisfies the far-field condition, and the −3 dB beam footprint is fully contained within the 300 × 300 mm^2^ sample. Because this quasi-monostatic setup captures the reflected wave, the raw transmission coefficient S_21_ recorded by the VNA is treated as the equivalent reflection coefficient S_11_ throughout our analysis. Using an identical bare aluminum plate as the full-reflection reference, the system was calibrated via the response method. An x-polarized plane wave was used for normal incidence, and the sample edges were wrapped with pyramidal absorbers to mitigate edge diffraction.

Figure 12b illustrates the measured signals and the derived absorptance. The bare aluminum plate (purple line) provides a baseline reflection of roughly −25 dB. To extract the true reflectance, the background must be eliminated. Because the VNA data is in the logarithmic dB scale, the normalized reflection in the dB domain (R_dB_) is obtained by subtracting the aluminum reference from the sample signal, R_dB_ = S_21,sample_ (dB) − S_21,Al_ (dB). This R_dB_ directly corresponds to the “Amount of attenuation” (green dashed line). The linear power reflectance R is then recovered via $R=10^{\frac{R_{\mathrm{dB}}}{10}}$, from which the absorptance (red line) is calculated as A ≈ 1 − R.

The measured signal exhibits a deep reflection dip at 11.8 GHz, yielding an absorptance near unity. This resonance shows a slight frequency shift compared to the simulated 10.68 GHz. Such shifts are common in practical microwave absorbers and arise from a combination of assembly and material factors. For instance, microscopic air gaps introduced during layer-by-layer assembly can alter the equivalent capacitance, while batch-to-batch deviations in PMMA permittivity and ITO sheet resistance can shift the resonant condition. Furthermore, the finite size (300 × 300 mm^2^) of the fabricated sample introduces edge truncation effects absent in the infinite periodic array model. Despite this shift, the structure maintains its broadband performance, with absorptance exceeding 90% across the entire 8.0–18.0 GHz band.

### 4.2. Reflection Shielding Effectiveness

Using the measured reflectance, the reflection shielding effectiveness (SE_R_) was calculated and is shown in Figure 12c. In the design band, SE_R_ remains well below 3.01 dB, confirming that the shielding mechanism of the covered solar panel is absorption-dominated. This marks the successful conversion of the solar panel from a reflection-type to an absorption-type shielding material.

### 4.3. Optical Transparency Test

The optical transmittance was characterized using an LH-221 spectrophotometer (Dongguan Laika Electronic Equipment Co., Ltd., Dongguan, China). Because the opaque solar panel substrate would completely block the light, the measurement was performed directly on the standalone ITO-PET-PMM layers to evaluate its intrinsic optical transparency. The measurement was focused on the visible wavelength range of 400–700 nm, which is a critical spectral region for the light absorption of GaAs and Ge-based solar cells. After baseline calibration, the transmittance was recorded by randomly measuring 10 distinct sampling points to avoid local inhomogeneities caused by the patterned layers.

As shown in Figure 12d, the measured transmittance varies between approximately 71% and 75%, yielding an average visible transmittance of approximately 73%. It should be emphasized that this result represents the optical transmittance of the absorber itself. These optical measurements strictly confirm the high visible transmittance of the standalone OTMMA layer, demonstrating its optical transparency while maintaining excellent microwave performance.

### 4.4. Fabrication Tolerance Analysis

**Figure 13:**
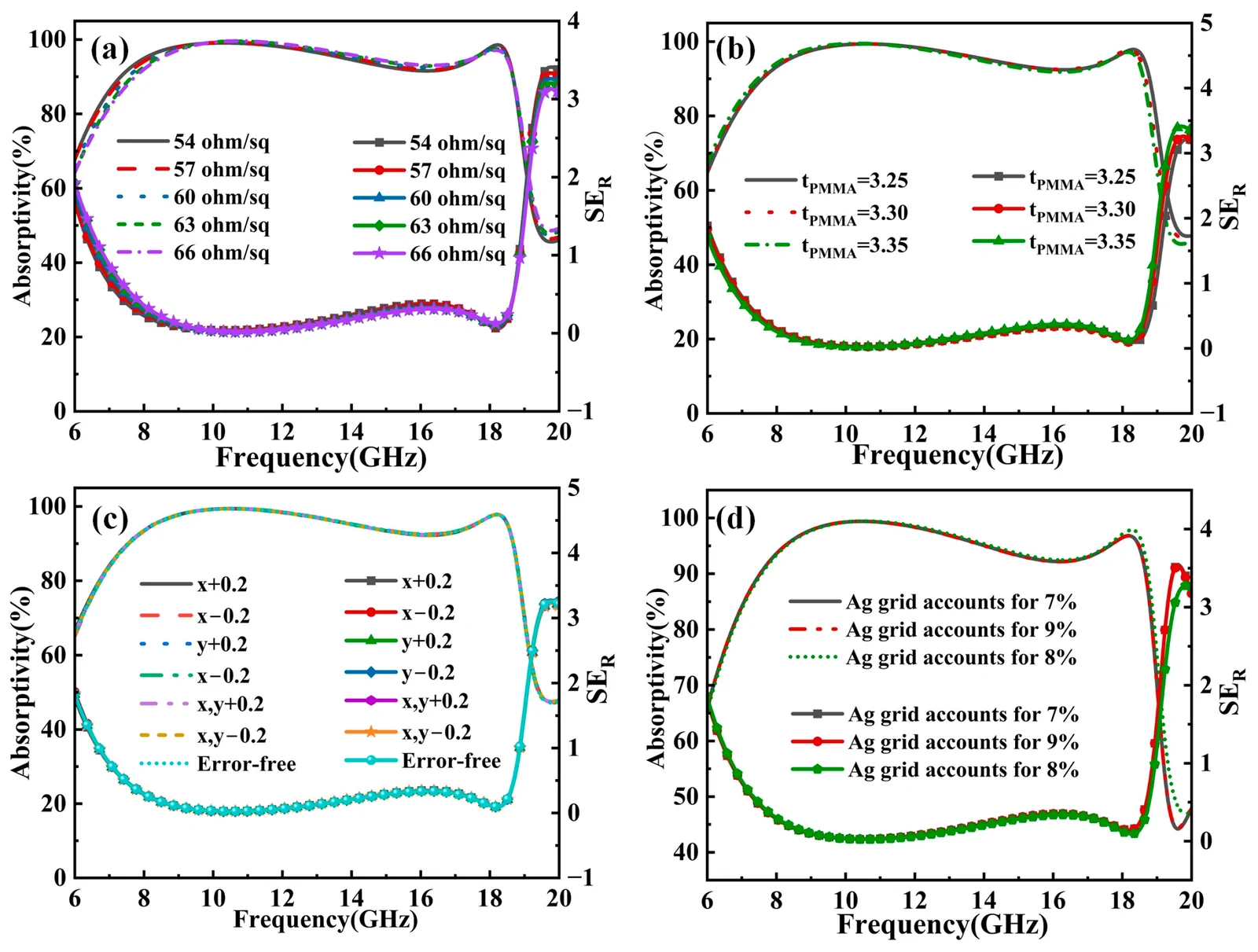
Simulated absorptance and SE_R_ under different fabrication tolerances: (**a**) ITO sheet resistance; (**b**) PMMA thickness; (**c**) Alignment errors in x- and y-directions; (**d**) Ag grid coverage ratio.

To establish the practical manufacturability of the proposed OTMMA, a quantitative parametric tolerance analysis was conducted via CST simulations. As illustrated in Figure 13, we evaluated the sensitivity of both the absorptance and the SE_R_ to four typical fabrication deviations. For the material properties, a ±10% deviation in the ITO sheet resistance (54 to 66 ohm/sq, Figure 13a) and a variation in the Ag grid coverage from 7% to 9% (Figure 13d) cause almost no degradation to the broad >90% absorption band. For the structural parameters, when the PMMA thickness fluctuates by ±0.05 mm (3.25 to 3.35 mm, Figure 13b) or the patterned layers suffer an alignment error of up to ±0.2 mm (Figure 13c), only negligible frequency shifts are observed at the high-frequency edge, while the overall broadband absorption remains largely intact. Crucially, across all evaluated tolerance ranges, the SE_R_ (dashed lines) consistently remains well below the 3.01 dB threshold across the entire design band. These results collectively confirm that the proposed OTMMA possesses excellent robustness to manufacturing deviations, ensuring reliable large-scale fabrication while preserving the desired absorption-dominated shielding mechanism.

## 5. Conclusions

In view of the metal-like characteristics of satellite solar panel, an optically transparent metamaterial absorber (OTMMA) was designed using the solar panel as the substrate. Within the 8–18 GHz band, the structure achieves an absorptance exceeding 90%, while the reflection shielding effectiveness (SE_R_) drops from over 15 dB to below 3.01 dB. This confirms the successful conversion of the shielding mechanism from reflection-dominated to absorption-dominated. Simulations performed on a finite 300 × 300 mm^2^ plate under normal-incidence, x-polarized conditions demonstrate that the monostatic RCS is reduced by more than 10 dB across the 6–20 GHz range, with a maximum reduction of 20.43 dB at 10.7 GHz. Additionally, the proposed OTMMA exhibits excellent angular stability and maintains an average visible transmittance of approximately 73%. By combining macroscopic equivalent parameters with microscopic field distributions, the underlying absorption mechanisms were systematically analyzed. Experimental measurements further verified the high absorption and optical transparency of the fabricated prototype, demonstrating its great potential for integrated electromagnetic purification and radar stealth applications on satellites.

## Data Availability

The original contributions presented in this study are included in the article. Further inquiries can be directed to the corresponding author.
